# Tick-borne encephalitis virus (TBEV) antibodies in animal sera – occurrence in goat flocks in Germany, longevity and ability to recall immunological information after more than six years

**DOI:** 10.1186/s12917-019-2157-5

**Published:** 2019-11-06

**Authors:** Christine Klaus, Ute Ziegler, Donata Hoffmann, Franziska Press, Christine Fast, Martin Beer

**Affiliations:** 1 Institute of Bacterial Infections and Zoonoses, Friedrich-Loeffler-Institut, Naumburger Str. 96a, 07743 Jena, Germany; 2 Institute of Novel and Emerging Infectious Diseases, Friedrich-Loeffler-Institut, Südufer 10, 17493 Greifswald, Insel Riems Germany; 3 Institute of Diagnostic Virology, Friedrich-Loeffler-Institut, Südufer 10, 17493 Greifswald, Insel Riems Germany

**Keywords:** Tick borne encephalitis, Animal sera, Virus neutralization test, *Flaviviridae*

## Abstract

**Background:**

TBE is an important tick-borne viral zoonosis in Europe and some parts of Asia. Humans can become infected by tick bite and in some cases also by consumption of nonpasteurized raw milk and raw milk products from ruminants. Serological investigations of milking flocks can help to assess the risk of TBEV infection for humans. 735 blood samples from 50 goat flocks from four federal states of Germany were tested by TBEV-VNT to assess a potential risk for TBEV infection.

There are some gaps in the knowledge about immunity in animals, for example with regard to the longevity of TBEV immunity. Two goats and two sheep were immunized and TBEV antibody titers could be detected for up to 7 years.

Furthermore, nothing is known about a possible long-lasting immunological memory that could quickly be reactivated by an additional contact to TBEV. Seven years after the first immunization two goats and two sheep as well as two naïve goats and two sheep were boostered and TBEV antibody titers followed.

**Results:**

Only one sample in each of the three states was TBEV-antibody positive (VNT), albeit with low titers. However, in Baden-Württemberg seven samples were positive, among them four goats of the same flock.

TBEV-antibody positive titers were detected in goats for up to 6 years and 10 months, in sheep for up to 4 years and 7 months.

Seven years after immunization a clear immunological recall occurred in response to administration of one dose of vaccine in two goats and two sheep.

**Conclusion:**

It can be concluded that in the tested flocks the risk of an alimentary TBEV infection was low. However, in one single flock a considerably higher risk must be assumed.

Antibody titers in goats and sheep can last very long after contact to TBEV, albeit at a low level. This should be taken into consideration in cases where the risk of an alimentary infection is assessed in a flock by serological investigations.

The immunological recall gives rise to the suspicion that the immunological memory after a first contact to TBEV lasts for many years, probably lifelong.

## Background

In Europe and some parts of Asia, Tick-borne encephalitis (TBE) is a very important viral tick-borne zoonosis [1], and the TBE virus (TBEV), a flavivirus, occurs in so-called “natural foci”, whose size can range from large to very small. TBEV circulates especially between small mammals as local hosts and ticks as arthropod vectors. A large variety of mammalian species may become infected, and human TBE infections are notifiable in most endemic countries. Despite the reported human cases, symptomatic infections are also known (but very seldom) in dogs [2], horses [3], monkeys [4], and in single cases in sheep, mouflons and goats [5–7].

In addition, seroconversion without any specific clinical signs of a TBEV infection is described in some livestock animal species such as cattle, small ruminants and pigs [8–11]. In previous studies, TBEV-specific antibody titers in both goats and sheep were detected for up to 28 months after immunization, and cross reactivity against other related pathogens was very low [12]. Furthermore, grazing animals have been shown to be suitable sentinels for the detection of TBEV foci by serological surveillance [9].

Especially the infection of ruminants is relevant for public health: in goats and sheep, and more rarely also in cattle, TBEV can be excreted via milk for several days after infection. Viraemia was observed between one and 5 days, TBEV was excreted via milk some days longer for two to 7 days in subcutaneously infected sheep [13], in subcutaneously infected cows TBEV could be detected for two to 4 days after infection in the blood and for three to 5 days in the milk [14]. After infection of a goat reported by van Tongeren [15], TBEV was detected in the blood from one to 5 days, from three to 8 days in the milk. Nearly the same results for sheep and a cow were reported by Grešíková and Rehacek after infection via tick bite [16]. Balogh et al. [17] detected TBEV in milk between eight and up to 19 days after experimental infection of goats. In the last 20 years, human cases of alimentary TBE have been reported from several countries including Hungary, Austria, Estonia, Slovakia [18–21] and very recently also in Germany [22].

The TBEV antibody status of a flock can help evaluate a possible risk of alimentary TBE caused by raw milk from this flock. In this study, (i) 50 goat flocks with 735 animals from the federal states of Bavaria (BY), Baden-Württemberg (BW), Lower Saxony (LS) and Mecklenburg-West Pomerania (MV) were evaluated with regard to TBEV antibodies to estimate the risk of TBEV infection in animals that can cause TBEV excretion via milk for several days.

As described above, knowledge about clinical symptoms, immune response and cross reactivity and longevity of antibody response as well as about the epidemiology of TBEV in animals has increased over the last years. However, some basic questions still remain unanswered or have been incompletely addressed. One relevant parameter is e.g. the longevity of TBEV-specific immunity. In our study, (ii) longevity was observed for more than 6 years. Furthermore, it is unknown whether contact to TBEV causes a long-lasting immunological memory that is reactivated quickly by any further contact to TBEV. Therefore, (iii) this possible recall of immunological information was tested by an additional immunization 5 years after the first TBEV contact.

## Results

### TBEV-antibody positive samples in the selected goat flocks

In the period from 2013 to 2015, we investigated 735 plasma samples from 50 goat flocks from four different federal states in Germany. The sample panel for the entire three-year period was available only from BY (see Table 1). The serological results are as follows: for MV from overall 205 samples in nine flocks only one goat was positive with a very low TBEV-titer (ND_50_ 1:10). A similarly low titer (ND_50_ 1:30) only was detectedin one goat from LS with overall 126 animals from nine flocks investigated and in one goat from BY (ND_50_ 1:30) with overall 230 animals from 21 flocks tested over 3 years. In contrast, the results from BW: in 2013, three goats out of 69 animals from a total of three herds were tested positive, with two goats from the same herd showing significantly higher titers (ND_50_ 1:160 and 1:320). The remaining goat had a titer of ND_50_ 1:20. Similar results could also be seen for 2015 in BW, where a total of 105 samples from eight herds were tested. Here, four positive goats from one herd were determined, which also had very high TBEV titers (ND_50_ 1:120, 1:120, 1:160 and 1:480), see Table 1.

**Table 1:** Origin and serological results of the goat samples

	2013	2014	2015	total
MV flocks	9	–	–	9
MV samples	205	–	–	205
MV TBEV pos.	1	–	–	1
MV SNT titer (ND_50_)	1:10			
LS flocks	2	7	–	9
LS samples	77	49	–	126
LS TBEV pos.	1	0	–	1
LS SNT titer (ND_50_)	1:30			
BW flocks	3	–	8	11
BW samples	69	–	105	174
BW TBEV pos.	3^a^	–	4^b^	7
BW SNT titer (ND_50_)	1:20, 1:160, 1:320		2 × 1:120, 1:160, 1:480	
BY flocks	12	8	1	21
BY samples	115	105	10	230
BY TBEV pos.	0	1	0	1
BY SNT titer (ND_50_)		1:30		

^a^two positive samples from the same flock

^b^all four positive samples from the same flock

*MV* Mecklenburg-West Pomerania, *LS* Lower Saxony, *BW* Baden-Württemberg, *BY* Bavaria

Before starting, positive sera and plasmas from goats were tested for their suitability by TBEV-VNT. No deviations between sera and plasmas were seen, and both starting materials could be used equally well (data not shown).

### Longevity of TBEV-specific antibody titers in immunized animals

In the previous study, titers increased in the vaccinated sheep and goats until 18 weeks after the first immunization and decreased over the following weeks to reach lower but still positive titers 28 months after vaccination [12]. Here in the following months, the titers further decreased but were detectable in goats for the whole observation period (6 years and 10 months). Overall, one sheep was tested positive up to 3 years and 6 months, the other one up to 4 years and 7 months.

TBEV antibody titers of these four multiple vaccinated animals and of four additional single vaccinated animals without previous contact to TBEV are shown in Table 2. VNT titers detected in sera of the animals immunized years before (multiple vaccinated) after one, two and 3 months ranged between 1:5 and 1:160. Generally, VNT titers in goat sera were higher when compared to titers detected in the sheep sera. In addition, the two goat sera revealed reactive TBEV antibody titers already 1 week after booster immunization. In contrast, three of the four naïve animals developed only low antibody titers (up to 1:15) after immunization, while one sheep did not display a positive TBEV antibody titer at all.

**Table 2:** Boost of TBEV-antibody titers (VNT) in pre-immunized goats and sheep^a^

time point after last immunization	multiple vaccinated				single vaccinated			
	goat 1	goat 2	sheep 1	sheep 2	goat 3	goat 4	sheep 3	sheep 4
1 week	1:40	1:10	< 1:5	< 1:5	< 1:5	< 1:5	< 1:5	< 1:5
1 month	1:113	1:80	1:160	1:160	1:15	1:7.5	1:10	< 1:5
2 months	1:80	1:28.2	1:15	1:160	1:15	< 1:5	< 1:5	< 1:5
3 months	1:28.2	1:80	1:5	1:28.2	1:7,5	1:5	< 1:5	< 1:5

^a^VNT titers of all animals < 1:5 before immunization

All results were obtained either with blood samples collected for other usages (see ethics statement) or with only eight animals kept in one flock at the FLI. At present animal samples cannot be replaced completely for these investigations. In the study the number of animals was minimized and all procedures (only subcutaneous immunization and collection of blood samples) were refined.

## Discussion

Our field study for the evaluation of goat flocks in Germany showed a TBEV seropositivity in single animal samples only. Most of the identified TBEV-reactive goats were found in Baden-Württemberg (11 flocks with 174 tested animals) with eight TBEV antibody-positive sera (4.6%). In Bavaria, Mecklenburg-West Pomerania and Lower Saxony only a single TBEV antibody positive sample could be detected (0.43, 0.49 and 0.78% of all tested samples, respectively). These results clearly show that even in grazing animals in the selected regions, contact to TBEV via tick bites is seldom and therefore the general risk of alimentary TBEV infection for humans is low. However, within a spatial TBEV natural focus the local risk of an alimentary TBEV infection can be remarkably higher. Since these foci can be very small, it is possible that only one ruminant flock is involved and that this particular flock has a high risk of TBEV infections. One single flock in Baden-Württemberg exhibited e.g. four of the eight TBEV antibody-positive samples. This is in accordance with our own findings in a previous study with 3590 sera from sheep and 3793 sera from goats in 2003 and 2006–2009 in seven federal states of Germany with in general low seroprevalences for the whole state. However, in single flocks in the same village seroprevalences between 0 and 43% were seen which confirmed the patchy pattern of TBEV [9]. Nearly the same differences between 2% (Mureş County) and 27.73% (Bihor County) seropositive sheep were found in Romania with a higher prevalence than in our investigated samples of 15.2% of all tested 519 samples in Romania [23]. A low overall seroprevalence was seen in Lithuanian domestic ruminants of 1.7% in eight out of 18 checked regions with high regional differences. Sheep from one region had the highest rate of 16% [24].

In a TBEV-positive case, it is highly recommended to test all milk and milk products from the affected flock for TBEV and to conduct further epidemiological investigations including anamnestic information about the purchase of animals from other regions with a known TBEV risk. Unfortunately, such information was not available for this study. A nationwide database of small ruminants, equally structured as for bovines, could help in this context to provide more background information for the veterinary authorities. A clearly worthwhile recommendation with regard to such a flock is to sell only pasteurized milk and milk products to avoid any cases of alimentary TBEV.

We could also show that reactive antibodies against TBEV last very long after immunization, and immunized goats displayed a positive reaction for nearly 7 years. In sheep, this time span was considerably shorter (3 years and 6 months and 4 years and 7 months). In our study highest titers were seen around 13 weeks after the first dose (all together four doses) of a vaccine produced for humans and based on TBEV strain Neudoerfl [11]. With three doses of a new vaccine candidate for veterinary use by Salát et al. [25] the highest titers were observed around 42 days after first immunization with higher VNT titers like in our study [11]. This vaccine based on TBEV strain Hypr and may cause even better immunity in animals like our vaccine for human use. This should be tested in further investigations.

Interestingly, even after years the small ruminants exhibited a clear immunological recall reaction as demonstrated by the increasing antibody titers in response to administration of one dose of vaccine. This contrasted very well the sero-reactions detected in the four naïve control animals with low or no antibody response after immunization with a single dose of the vaccine.

In this study, the progress of TBEV antibody titers reflects the situation after immunization with an inactivated vaccine, however natural infection may result in relatively similar humoral immune responses, as already shown for horses and dogs [2, 3]. However, the duration of immunity could be different between booster-vaccinated animals and animals after natural TBEV-infection. A fact which should be also taken into consideration.

## Conclusions

TBEV-specific antibodies in grazing animals are not widespread, however, in some single flocks a remarkable number of seropositive animals indicate contact of the flock to TBEV. Consequently, a high risk of alimentary TBEV from non-pasteurized milk and milk products must be assumed in these flocks. In these cases epidemiological investigations like testing ticks from the meadows used by the flock for TBEV-RNA, avoiding meadows with TBEV-positive ticks and strict pasteurization of the milk before consumption are highly recommended.

We could also show that immunized goats and sheep had long-lasting TBEV-specific antibody titers, in our study between 3 years and 6 months and 6 years and 10 months. A boost immunization years after the first immunization demonstrated efficient immunological recall reactions.

Finally, it can be concluded that for organic farms selling fresh milk and milk products to private consumers the immunization of milking goats and sheep also could be an alternative to minimize the risk of an alimentary TBEV-infection.

## Methods

### Collection of samples

#### Samples from goat flocks

Between 2013 and 2015, blood samples (in particular plasma) were collected from a variety of goat breeds raised in nearly all federal states of Germany (excluding the cities Berlin, Hamburg and Bremen). The blood samples from four different federal states selected for this project were prioritized based on the continuous annual occurrence of TBEV in ticks and humans in southern Germany and recent cases of alimentary raw milk infections in humans [22]. In contrast, TBEV detection in ticks in central and northern Germany is rather sporadic and more locally restricted. All examined goat samples have in common, that contact of the animals with grassland and pastures was possible. Therefore, our extracted panel of 735 blood samples focused on the federal states BY, BW, LS and MV. More details with regard to the number of goat flocks and samples for each federal state and for each year are summarized in Table 1. All animals remained in their flocks of origin. No anaesthesia or euthanasia methods were used.

#### Sera collected to analyse the longevity of the humoral immune response

Two goats and two sheep were immunized four times with FSME-IMMUN Erwachsene (Baxter Deutschland GmbH, Unterschleißheim, Germany), a vaccine available for humans, as described by Klaus et al. [11], at weeks 0, 1, 3 and 11. The vaccine contains inactivated TBEV, strain Neudoerfl. At each immunization one dose of 0.5 ml containing 2.4 μg inactivated virus antigen was injected subcutaneously per goat and sheep. This dose is also used routinely for adult humans according to the manufacturer’s vaccination recommendations. All animals were observed for clinical signs of illness or allergic reactions as well as for a potential increase of body temperature at least 2 days before and 2 days after immunization. Neither clinical nor allergic signs were observed, and body temperature was normal at all times. According to the manufacturer’s instructions three immunizations were recommended in humans for a good immune response. However, in previous studies single animals showed delayed reactivity compared to the others and needed four immunizations to develop TBEV specific antibodies. Therefore, it was decided to immunize all animals four times [11]. TBEV antibody titers were traced over a period of 6 years and 10 months.

In order to check a possible recall of immunological information after nearly 7 years, these individual animals were booster immunized with one dose of “FSME-IMMUN Erwachsene” (Baxter Deutschland GmbH, Germany) subcutaneously, and as an additional control, two naïve goats and sheep were also immunized with one dose of the same vaccine. After (booster) immunization, TBEV titers weretraced for 3 months. All eight animals were kept in the same flock at the Friedrich-Loeffler-Institut (FLI) without pathogen contact in a natural TBEV focus. These animals were owned by the FLI and lived in one flock grazing on FLI owned meadows, with access to hay and concentrated feed stuff. No anaesthesia or euthanasia methods were used in any of the flocks. All animals were female, at the beginning of the first immunization between eight to 10 months old, at the time of boost immunization 7 years old, with a weight between 83 and 118 kg. Animal health status was checked daily. No health problems occurred during the whole study period.

All animal experiments were conducted at a high standard of veterinary care and were approved by the competent authorities.

### Test systems

#### TBEV specific serology

For the detection of TBEV antibodies we analysed all plasma samples from goats in a specific virus neutralization test (VNT) by using TBEV strain “Neudoerfl” (kindly provided by F. Hufert, Institute for Virology, Göttingen Germany; GenBank accession no. U27495).

The VNT was performed as already described by Seidowski et al. [26] and Ziegler et al. [27], only the virus strain (TBEV) was modified. Briefly, all samples were run in duplicate and at a starting plasma dilution of 1:10 and a virus concentration of 100 TCID_50_/well. The applied cells were Vero B4 (Collection of Cell Lines in Veterinary Medicine, Friedrich-Loeffler- Institut, Germany), cytopathic effects were revealed 6–7 days postinfection, and VNT titers were calculated. The neutralizing antibody titers (ND_50_) were expressed at the reciprocal of the plasma dilution that still inhibited > 50% of cytopathogenic effect, calculated according to the Behrens-Kaerber method. Plasma samples with ND_50_ values above 10 were determined as positive; samples with a lower titer than 10 were determined as negative.

Sera of immunized goats and sheep from the second and third part of this study were examined by virus neutralization test as described previously [28], virus strain Langat was used in the test. Here, serum samples with ND_50_ values above 1:5 were determined as positive; samples with a lower titer were classified as negative.

## Data Availability

The datasets used and/or analysed during the current study are available from the corresponding author on reasonable request.

## Abbreviations

BMEL: Federal Ministry of Food and Agriculture (Bundesministerium für Ernährung und Landwirtschaft)
BW: Baden-Württemberg
BY: Bavaria
DZIF: German Center for Infection Research (Deutsches Zentrum für Infektionsforschung)
FLI: Friedrich-Loeffler-Institute
LALLF: State Institution for Agriculture, Food Safety and Fishing (Landesamt für Landwirtschaft, Lebensmittelsicherheit und Fischerei), Mecklenburg-West Pomerania
LS: Lower Saxony
MV: Mecklenburg-West Pomerania
TBE: Tick-borne encephalitis
TBEV: Tick-borne encephalitis virus
TiHo: University of Veterinary Medicine Hannover (Stiftung Tierärztliche Hochschule Hannover)
TLLV: Thuringean State Institution for Food Safety and Consumer Protection (Thüringer Landesamt für Lebensmittelsicherheit und Verbraucherschutz), Bad Langensalza
VNT: Virus neutralization test

## References

[1] Süss J (2011). Tick-borne encephalitis 2010: epidemiology, risk areas, and virus strains in Europe and Asia – an overview. Ticks Tickborne Dis.

[2] Leschnik MW, Kirtz GC, Thalhammer JG (2002). Tick-borne encephalitis (TBE) in dogs. Int. J. Med. Microbiol..

[3] Rushton JO, Lecollinet S, Hubálek Z, Svobodová P, Lussy H, Nowotny N (2013). Tick- borne encephalitis virus in horses, Austria, 2011. Emerg. Infect. Dis..

[4] Süss J, Gelpi E, Klaus C, Bagon A, Liebler-Tenorio EM, Budka H (2007). Tickborne encephalitis in naturally exposed monkey (*Macaca sylvanus*). Emerg. Infect. Dis..

[5] Böhm B, Schade B, Bauer B, Hoffmann B, Hoffmann D, Ziegler U, et al. Tick-borne encephalitis in a naturally infected sheep. BMC Vet. Res. 2017. 10.1186/s12917-017-1192-3. 10.1186/s12917-017-1192-3 PMC5567888 28830430

[6] Bagó Z, Bauder B, Kolodziejek J, Nowotny N, Weissenböck H (2002). Tickborne encephalitis in a mouflon (*Ovis ammon musimon*). Vet. Rec..

[7] Zindel W, Wyler R. Zeckenenzephalitis bei einer Ziege im Prättigau [Tick-borne encephalitis in a goat in Prättigau, Switzerland]. Schweiz. Arch. Tierheilkd.. 1983;125:383–386 (in German, with English abstract). 6612291

[8] Leutloff R, Nübling M, Neumann-Haefelin D, Rieger MA (2006). Cows as indicators for tick-borne encephalitis (TBE) endemic regions: suitability of testing for antibodies in serum and milk. Int. J. Med. Microbiol..

[9] Klaus C, Beer M, Saier R, Schau U, Moog U, Hoffmann B (2012). Goats and sheep as sentinels for tick-borne encephalitis (TBE) virus - epidemiological studies in areas endemic and non-endemic for TBE virus in Germany. Ticks Tickborne Dis..

[10] Grešíková M, Sekeyová M, Stúpalová S, Nečas S (1975). Sheep milk-borne epidemic of tick-borne encephalitis in Slovakia. Intervirology.

[11] Klaus C, Beer M, Saier R, Schubert H, Bischoff S, Süss J (2011). Evaluation of serological tests for detecting tick-borne encephalitis virus (TBEV) antibodies in animals. Berl Munch Tieraerztl Wochenschr..

[12] Klaus Christine, Ziegler Ute, Kalthoff Donata, Hoffmann Bernd, Beer Martin (2014). Tick-borne encephalitis virus (TBEV) – findings on cross reactivity and longevity of TBEV antibodies in animal sera. BMC Veterinary Research.

[13] Grešíková M (1958). Recovery of the tick-borne encephalitis virus from the blood and milk of subcutaneously infected sheep. Acta Virol..

[14] Grešíková M (1958). Excretion of the tick-borne encephalitis virus in the milk of subcutaneously infected cows. Acta Virol..

[15] Van Tongeren HA (1955). Encephalitis in Austria. IV. Excretion of virus by milk of the experimentally infected goat. Arch. Gesamte Virusforsch..

[16] Grešíková M, Rehacek J (1959). Isolation of the tick-borne encephalitis virus from the blood and milk of domestic animals (sheep and cow) after infection by ticks of the family *Ixodes ricinus*. Arch. Gesamte Virusforsch..

[17] Balogh Z, Egyed L, Ferenczi E, Bán E, Szomor K, Takács M, Berencsi G (2012). Experimental infection of goats with tick-borne encephalitis virus and the possibilities to prevent virus transmission by raw goat milk. Intervirology.

[18] Balogh Z, Ferenczi E, Szeles K, Stefanoff P, Gut W, Szomor K (2010). Tick-borne encephalitis outbreak in Hungary due to consumption of raw goat milk. J. Virol. Methods.

[19] Holzmann H, Aberle SW, Stiasny K, Werner P, Mischak A, Zainer B (2009). Tick-borne encephalitis from eating goat cheese in a mountain region of Austria. Emerg. Infect. Dis..

[20] Kerbo N, Donchenko I, Kutsar K, Vasilenko V (2005). Tick-borne encephalitis outbreak in Estonia linked to raw goat milk. Euro Surveill..

[21] Kohl I, Kozuch O, Elecková E, Labuda M, Zaludko J (1996). Family outbreak of alimentary tick-borne encephalitis in Slovakia associated with a natural focus of infection. Eur. J. Epidemiol..

[22] Brockmann SO, Oehme R, Buckenmaier T, Beer M, Jeffery-Smith A, Spannenkrebs M, et al. A cluster of two human cases of tick-borne encephalitis (TBE) transmitted by unpasteurised goat milk and cheese in Germany, may 2016. Euro Surveill. 2018. 10.2807/1560-7917.ES.2018.23.15.17-00336. 10.2807/1560-7917.ES.2018.23.15.17-00336 PMC6836198 29667575

[23] Salat Jiri, Mihalca Andrei D., Mihaiu Marian, Modrý David, Ruzek Daniel (2017). Tick-Borne Encephalitis in Sheep, Romania. Emerging Infectious Diseases.

[24] Juceviciene Aurita, Zygutiene Milda, Leinikki Pauli, Brummer-Korvenkontio Henrikki, Salminen Mika, Han Xiuqi, Vapalahti Olli (2005). Tick-borne encephalitis virus infections in Lithuanian domestic animals and ticks. Scandinavian Journal of Infectious Diseases.

[25] Salát Jiří, Formanová Petra, Huňady Milan, Eyer Luděk, Palus Martin, Ruzek Daniel (2018). Development and testing of a new tick-borne encephalitis virus vaccine candidate for veterinary use. Vaccine.

[26] Seidowski Diana, Ziegler Ute, von Rönn Jan A.C., Müller Kerstin, Hüppop Kathrin, Müller Thomas, Freuling Conrad, Mühle Ralf-Udo, Nowotny Norbert, Ulrich Rainer G., Niedrig Matthias, Groschup Martin H. (2010). West Nile Virus Monitoring of Migratory and Resident Birds in Germany. Vector-Borne and Zoonotic Diseases.

[27] Ziegler Ute, Jöst Hanna, Müller Kerstin, Fischer Dominik, Rinder Monika, Tietze Dieter Thomas, Danner Klaus-Jürgen, Becker Norbert, Skuballa Jasmin, Hamann Hans-Peter, Bosch Stefan, Fast Christine, Eiden Martin, Schmidt-Chanasit Jonas, Groschup Martin H. (2015). Epidemic Spread of Usutu Virus in Southwest Germany in 2011 to 2013 and Monitoring of Wild Birds for Usutu and West Nile Viruses. Vector-Borne and Zoonotic Diseases.

[28] Klaus C, Hoffmann B, Moog U, Schau U, Beer M, Süss J. Can goats be used as sentinels for tick-borne encephalitis (TBE) in non-endemic areas? Experimental studies and epizootiological observations. Berl Munch Tieraerztl Wochenschr. 2010;123:441-5. 21141272

